# Leveraging electronic health record data for endometriosis research

**DOI:** 10.3389/fdgth.2023.1150687

**Published:** 2023-06-05

**Authors:** Nadia Penrod, Chelsea Okeh, Digna R. Velez Edwards, Kurt Barnhart, Suneeta Senapati, Shefali S. Verma

**Affiliations:** ^1^ College of Agriculture and Life Sciences, Texas A&M University, College Station, TX, United States; ^2^ Department of Pathology and Laboratory Medicine, Perelman School of Medicine, Philadelphia, PA, United States; ^3^ Department of Obstetrics and Gynecology, Vanderbilt University, Nashville, TN, United States; ^4^ Department of Obstetrics and Gynecology, Perelman School of Medicine, University of Pennsylvania, Philadelphia, PA, United States

**Keywords:** reproductive health, women’s health, electronic health records—EHR, endometriosis, obstetric & gynecologic

## Abstract

*Endometriosis* is a chronic, complex disease for which there are vast disparities in diagnosis and treatment between sociodemographic groups. Clinical presentation of endometriosis can vary from asymptomatic disease—often identified during (in)fertility consultations—to dysmenorrhea and debilitating pelvic pain. Because of this complexity, delayed diagnosis (mean time to diagnosis is 1.7–3.6 years) and misdiagnosis is common. Early and accurate diagnosis of endometriosis remains a research priority for patient advocates and healthcare providers. Electronic health records (EHRs) have been widely adopted as a data source in biomedical research. However, they remain a largely untapped source of data for endometriosis research. EHRs capture diverse, real-world patient populations and care trajectories and can be used to learn patterns of underlying risk factors for endometriosis which, in turn, can be used to inform screening guidelines to help clinicians efficiently and effectively recognize and diagnose the disease in all patient populations reducing inequities in care. Here, we provide an overview of the advantages and limitations of using EHR data to study endometriosis. We describe the prevalence of endometriosis observed in diverse populations from multiple healthcare institutions, examples of variables that can be extracted from EHRs to enhance the accuracy of endometriosis prediction, and opportunities to leverage longitudinal EHR data to improve our understanding of long-term health consequences for all patients.

## Introduction

Electronic health records (EHRs) are digital repositories that chronicle the practice of medicine. They include established standards of care and the documented intuition and *ad hoc* methods clinicians rely on to diagnose and treat complex, heterogeneous diseases. Because multiple aspects of patient care (including time course, the severity of signs and symptoms, comorbidities, and treatments) are documented, the information contained within EHRs can be used to design large-scale, retrospective studies to establish patterns predictive of a complex disease, which can then be used prospectively to identify patients at risk of the disease before a formal diagnosis is made. Endometriosis is a disease for which EHR-based research may be particularly valuable, as this disease is often difficult to diagnose and manage given that the patients suffer from a wide range of symptoms (1). EHRs can provide a rich source of information on the symptoms, treatments, and outcomes associated with endometriosis, allowing researchers to better understand the disease and develop new approaches for diagnosis and treatment. Furthermore, because endometriosis is a complex disease that affects multiple aspects of a patient's health, EHR-based research can help to identify patterns and risk factors that traditional study designs may miss. Some of the largest epidemiological studies in endometriosis use Nurses Health Study II data, prospective cohort study to collect reproductive and lifestyle data from women via self-administered questionnaires (1–3). Prospective cohort-based studies are undoubtedly useful, however, recent rise in the use of structured and unstructured EHR data offers an opportunity for capturing large diverse patient populations for endometriosis research. For instance, one of the largest real-world evidence-based studies for evaluating the economic burden of endometriosis highlighted the Truven Health MarketScan commercial database which includes data extracted from EHR to understand healthcare utilization (4). This study concluded that endometriosis patients encounter with healthcare system more often than non-endometriosis patients. With such high utilization of healthcare by endometriosis patients, it is arguably imperative to utilize the same resource for clinical research. However, EHR in biomedical and clinical research for endometriosis is still under-utilized.

Endometriosis is an estrogen-dependent, chronic inflammatory disease of the female reproductive system (5, 6). Clinical presentation can vary from asymptomatic disease—often identified during (in)fertility consultations—to dysmenorrhea and debilitating pelvic pain (7–10). Because of this complexity, diagnosing endometriosis can be difficult; gynecologists have the highest diagnostic performance, but even patients who report symptoms to a gynecologist have a mean time to diagnosis of 1.7–3.6 years (11, 12).

Endometriosis develops when endometrial cells travel from the uterine cavity to ectopic sites outside the uterine lining, embed and grow into endometriosis lesions (5, 10). These lesions respond to hormonal signals during menstrual cycles or pregnancy, proliferating in the presence of estrogens and androgens and receding in the presence of progesterone. Endometriosis is a progressive condition, and untreated endometriosis lesions cause inflammation, leading to scar tissue that disfigures the pelvic anatomy and results in chronic pelvic pain, dyspareunia, and infertility. Patients with endometriosis have significantly higher all-cause healthcare costs and diminished quality of life, social well-being, and productivity (13–15).

Pelvic laparoscopic surgery is required to definitively diagnose and treat endometriosis. Although laparoscopic excision or ablation of lesions may increase viable intrauterine pregnancy rates, there is limited data to demonstrate that surgery increases live birth rates, and there is no definitive evidence that it reduces pain (16). Laparoscopy is not universally available, nor is it without procedure-related risks, and approximately 50% of endometriosis diagnoses are assumed without laparoscopic surgery (12).

It is commonly reported that 10% of menstruating adolescent and adult females have endometriosis (5, 6). But there is tremendous variation in the reported incidence and prevalence of endometriosis based on the study population, specialty of the diagnosing clinician, and diagnostic procedures (12, 17–20). As study populations consist of mostly White and Asian women, the prevalence of endometriosis is especially underestimated in women of other races and ethnicities (21, 22). Because pelvic pain is a nonspecific symptom and societal or cultural pressure attempts to normalize or diminish the significance of menstrual pain, endometriosis is frequently misdiagnosed as a more widely recognized genitourinary or gastrointestinal disease, such as pelvic inflammatory disease, urinary tract infection, or irritable bowel syndrome. Delay of diagnosis and misdiagnoses may lead to adverse outcomes, including delayed care, reoperation, surgical complications, and intraoperative injuries (22).

Early and accurate diagnosis of endometriosis continues to be a research priority for patient advocacy groups and healthcare professionals (23). EHRs, as a source of real-world, big data, are a largely untapped resource for endometriosis research that can be mined to learn which heterogeneous patterns of health history and symptoms manifest in an endometriosis diagnosis and thus aid in the establishment of screening guidelines to help clinicians efficiently and effectively recognize and diagnose the disease (24). Endometriosis is a condition often mistaken or misdiagnosed, as its tell-tale symptoms are not uncommon main other ailments that afflicting women. EHR are rich sources of clinical health data that offer insight into a patient's health experience. Many women with endometriosis who visit health providers prior to an official diagnosis often state the presence of symptoms such as abdominal pain, and heavy/irregular menstrual bleeding as recorded in the EHR. Use of the EHR by integrating structural, clinical notes, and patient-reported outcomes may even offer a retrospective approach to determining when symptoms started to better understand the symptomatology and onset of the condition.

## Advantages of electronic health record data

EHRs are digital records of patient health data collected in real-time at the point of care and maintained by healthcare providers. Currently, there is a shift toward EHR data in epidemiological studies. Given the success of using EHR for epidemiological research in many other disease conditions such as diabetes, cardiovascular disease, cancer, mental health disorders, uterine fibroids, and many other conditions, we argue that it would be facilitative for endometriosis research. A study published by Ambrosy et al. leveraged EHR data to identify specific patterns or trends in the presentation of heart failures, such as changes in vital signs or laboratory values, that may indicate the onset of acute decompensation. This EHR-based epidemiological study was able to accurately identify 90% of individuals who are experiencing acutely decompensated heart failure (25). Another example is a study published Yu et al. in 2018, which used EHR data to investigate the epidemiology of uterine fibroids in the United States. The study found that the prevalence of uterine fibroids among black women was highest than the other populations (18.5%), and that the incidence of the disease was disproportionally higher in young women (26). These studies demonstrate the broad applicability of EHR data for epidemiological research and highlight the potential of this type of data to provide valuable insights into the epidemiology of various conditions.

Patient health data are primarily documented and stored in two formats: (1) structured data that relies on a controlled vocabulary, including demographics, diagnostic codes [e.g., International Classification of Diseases (ICD) codes], procedure codes, laboratory test results, and medications; and (2) unstructured data in the form of uncontrolled free text, including clinician notes and imaging reports (27, 28). EHRs were developed to track and manage patient care and billing, but it quickly became apparent that these repositories would be an invaluable source of data for clinical research (29). Observational data from EHR is used in many emulated clinical trials to provide a more realistic and representative view of diagnosis and treatment in the real world (28). EHR can also be useful for endometriosis research through emulated clinical trials by providing a large and diverse dataset for researchers to analyze. This can help researchers to identify patterns and trends in endometriosis diagnosis and treatment and to develop and test new diagnostic and treatment approaches. EHR can also facilitate the recruitment of study participants, as it allows researchers to easily identify and contact individuals with endometriosis who may be interested in participating in a clinical trial. EHR data gives us the advantage of including patient participants with endometriosis. This data, combined with existing longitudinal data in the EHR, allows us to also predict long-term outcomes. This can be considered a paradigm change as we do not have to follow patients as strategically as in traditional longitudinal studies, and we can still look at the data more retrospectively.

### Large sample sizes and diverse populations

EHRs represent the patient base of local or regional healthcare systems. In the United States, 89.9% of office-based physicians use EHRs to manage patient data (30). Unlike clinical trials, EHRs do not have rigid exclusion criteria or other barriers limiting participation, so these repositories cover larger and more diverse populations than other clinical datasets and are more likely to accurately represent the source population demographically. Some EHRs are linked to biobanks that include genotype arrays or whole exome or whole genome sequencing data that can be used to elucidate the molecular mechanisms and pathophysiology underlying complex diseases. The UKBiobank, All of Us, Geisinger Mycode, BioVU, BioMe, and Penn Medicine Biobanks are examples of EHR-linked biobanks that have consented and recruited over 2 million patients worldwide (31–36).

Large sample sizes are important in studies of endometriosis because, as evidenced by the complex and heterogeneous clinical presentations, endometriosis is not a single disease entity (37, 38). Instead, it is an amalgamation of various symptoms and clinical diagnostic criteria. Subtyping at the molecular level is an active area of endometriosis research, and biomarkers are a coveted diagnostic tool for this disease. Subtyping endometriosis by clinical variables such as risk factors, symptomology, and treatment response profiles are important to provide the context necessary to interpret diagnostic biomarkers and establish screening guidelines. EHRs capture these data at the population level, thereby providing adequate sample sizes to allow meaningful stratification by clinical variables. Algorithms can be trained to identify subgroups by finding combinations of unique data elements (39, 40). For endometriosis, these data elements may include family history, parity, and painful or irregular menstruation. Additionally, because these algorithms are built using population-based data, there will be subtypes that generalize to all patients, regardless of ancestry, sex, or socioeconomic status.

Currently, the endometriosis literature is heavily biased toward studies that represent White and Asian women. There is scarce literature on delayed or missed endometriosis diagnoses in Black or Hispanic women, and almost nothing is known about endometriosis in transgender men, for whom there are only two published studies evaluating disease incidence and severity (21, 41, 42). Consequently, underrepresentation in research may limit women of color from benefitting from research findings and novel treatments (42). Evidence to determine if ancestry or race plays a role in the type, severity, or prevalence of the disease is also limited (41, 43–46). For example, endometriosis implants on the uterus are considered atypical (most documented lesions are found in the ovaries, bladder, or colon), but a retrospective chart review study revealed that 93% of African American women who underwent laparoscopy had uterine implants (43).

African American women also have lower rates of laparoscopically confirmed endometriosis diagnoses when compared to their White counterparts. This may partly explain why Black women are less likely to be diagnosed with endometriosis than White women (21, 35). It is also the case that White women are less likely to be diagnosed with endometriosis than Asian women (41, 47, 48). Epidemiological and genomic studies of endometriosis mostly focus on European or East Asian populations due to the lack of availability of data on other ancestry individuals (1, 49, 50). However, when we searched the TriNetX database, a global source of EHR data, we found that the prevalence of endometriosis based on ICD, 10th revision (ICD-10) codes was 4.2% for Black women, 3.7% for White women, and 3.0% for Asian women (Table 1). Given that endometriosis is underdiagnosed, lower than 10% prevalence is expected. However, data from Table 1 demonstrate the capabilities of EHR in extracting individuals of non-European ancestry patient participants for endometriosis research. Designating implantation sites as atypical when they are common in a subset of patients and reporting inconsistencies in disease prevalence by ancestry are the result of data gaps that distort findings and perpetuate unknowns about the pathophysiology of endometriosis. EHRs can fill these data gaps with comprehensive and inclusive real-world data that would otherwise never be collected because of the cost and logistics (17, 51).

**Table 1:** Trinetx network, ICD10-based endometriosis prevalence among patient encounters with a gynecologist.

	Patients, No (%)		
	Encounter with gynecologist (*n* = 2,443,350)	Encounter with gynecologist and endometriosis ICD10 code (*n* = 88,860)	Prevalence of Endometriosis (based on diagnosis codes)
**Age, years**			
15–24	771,017 (31.6)	34,220 (38.5)	4.4%
25–34	649,053 (26.6)	33,521 (37.7)	5.2%
35–44	582,766 (23.9)	15,016 (16.9)	2.6%
45–54	325,648 (13.3)	4,742 (5.3)	1.5%
55–64	92,132 (3.8)	1,049 (1.2)	1.1%
65+	22,734 (0.9)	312 (0.4)	1.4%
**Race**			
American Indian or Alaska Native	6,757 (0.3)	341 (0.4)	5.0%
Asian	73,444 (3.0)	2,235 (2.5)	3.0%
Black or African American	425,279 (17.4)	18,018 (20.3)	4.2%
Native Hawaiian or Other Pacific Islander	2,425 (0.1)	162 (0.2)	6.7%
White	1,539,846 (63.0)	57,378 (64.6)	3.7%
Unknown	395,599 (16.2)	10,726 (12.1)	2.7%
**Ethnicity**			
Hispanic	309,589 (12.7)	10,312 (11.6)	3.3%
Unknown	758,216 (31.0)	21,096 (23.7)	2.8%

Data: TriNetX network, April 11, 2022.

### Rich phenotyping

Data in the EHR can be found in both structured and unstructured forms. Structured data, as its name denotes, has a structure in that it follows protocols and is readily extractable by both programs and humans. On the other hand, unstructured data is not readily extractable by programs and may rely on tools like natural language processing. Structured data is usually found in databases, while unstructured data is found to be freeform or requires nuanced interpretation. Clinical notes and images are examples of unstructured data. In contrast, examples of structured data include but are not limited to, diagnosis identifiers (ICD codes, CPT codes, SNOMED-CT codes, etc.) and laboratory measurements and vitals such as BMI, weight, and height. For EHR data to be useful for research, rigorous phenotyping is required to identify valid disease-specific cases and controls to ensure high-quality study populations (28, 52). The most vetted EHR-derived phenotyping algorithms rely on ICD codes, medications, concepts identified in clinical notes using natural language processing, clinical procedural terminology (CPT) codes, and laboratory test results (53). These algorithms, such as those deposited in the PheKB database, have a high positive predictive value and are portable across different healthcare organizations. PheKB is a database funded by NHGRI through the electronic Medical Records and Genomics Network. This database act as a resource and collaborative environment to deposit and test EHR-based algorithms to define phenotypes that can be used for research.

A validated phenotyping algorithm for endometriosis, implemented in OHDSI (the Observational Health Data Sciences and Informatics Collaborative), incorporates diagnostic codes and endometriosis-related procedure codes for visualizing lesions, including those for pelvic laparoscopy and pelvic imaging. When applied retrospectively to EHR data, the algorithm performed with nearly 80% accuracy and had a 70% sensitivity for avoiding false-positive cases (54). This means that after manual chart reviews, 80% of patients who were true cases for endometriosis were identified, and 70% of patients that were not cases for endometriosis were excluded from the cohort. However, recall and prevalence of endometriosis in EHRs are underestimated when phenotyping algorithms rely on diagnostic codes and procedures alone because there are no standard screening protocols for endometriosis to generate a consistent, structured data element (7, 8). For diseases like endometriosis that are often underdiagnosed or misdiagnosed, the clinical notes are critical for phenotyping.

The underrepresentation of chronic medical conditions when relying on diagnostic codes alone has been previously described. For example, when relying solely on ICD-10 codes to identify patients with nonalcoholic fatty liver disease (NAFLD) in EHRs and insurance claims databases, disease prevalence was underestimated by 40% (55). This contrast with type 2 diabetes, a common comorbidity of NAFLD, which was identified using ICD-10 codes with an accuracy exceeding 95% in the same study. When researchers used an algorithm including data from the clinical chart notes and triglyceride levels in addition to diagnostic codes, they were able to close this gap and identify more NAFLD patients with a high probability (56).

**Figure 1:**
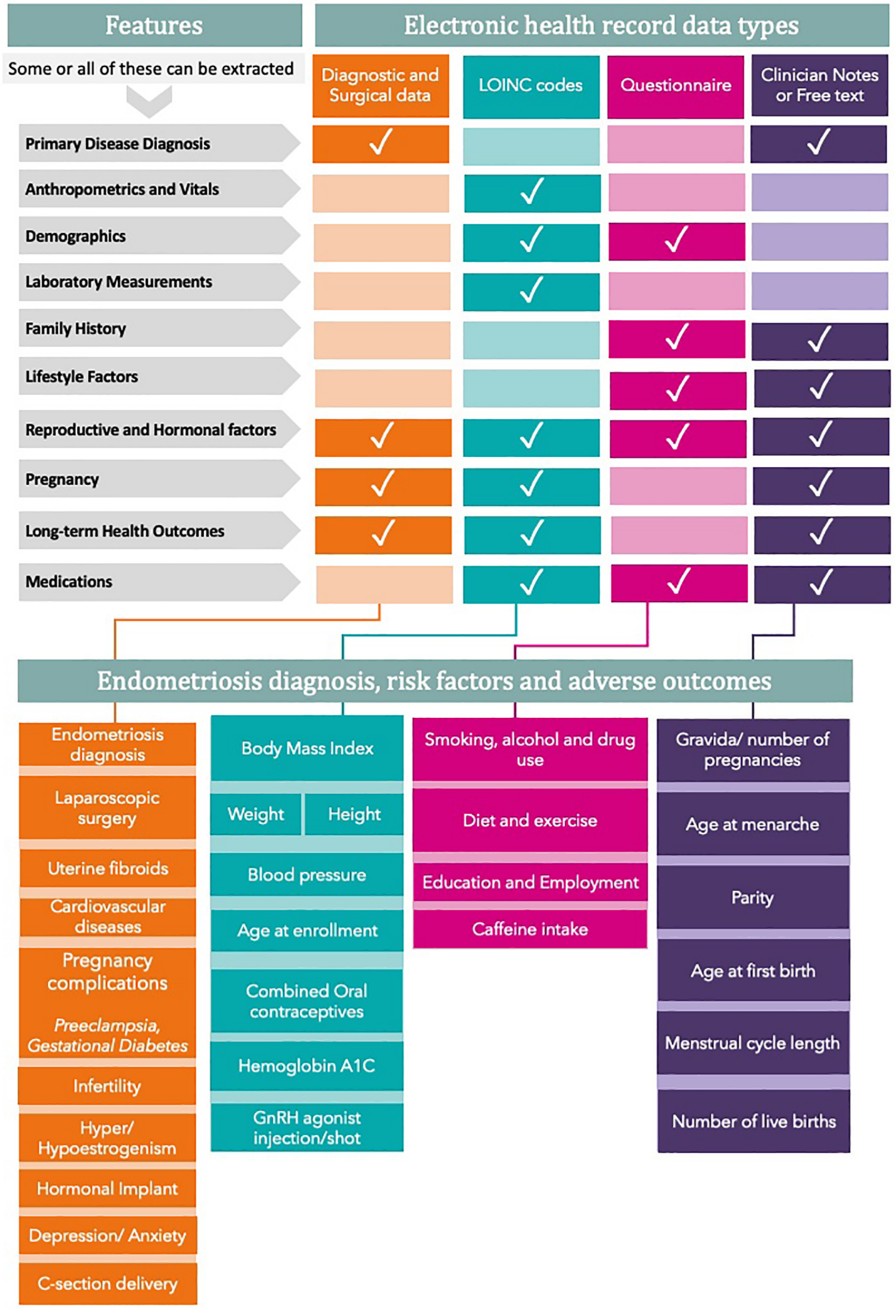
Risk factors that can be extracted from EHR for endometriosis research. All variables are divided into features that can be extracted from various EHR data types. The bottom panel clusters all variables into four main groups. GnRH refers to gonadotrophin-releasing hormone.

The ability to phenotype patients based on combinations of data elements that do not have to be defined *a priori* is a significant strength of real-world EHR data. This type of data-driven phenotyping is becoming increasingly more sophisticated, taking into account probabilities and disease severity instead of binary labels and recognizing the importance of timing and relationships between events (57, 58). For endometriosis, this involves incorporating risk factors, such as family history, parity, menstrual irregularities, and other known information documented in EHRs (Figure 1), to identify heterogeneous patterns that define subtypes. This type of data-driven phenotyping can also be used for risk stratification to facilitate early detection and reduce delays in diagnosis.

### Longitudinal data

EHRs track patients over time as they move between physicians and clinics within a health care system, and if a patient leaves a health care system, EHRs across systems can be linked (29). This generates clinical data with a temporal component broadening the research questions that can be addressed to include exploring the relationships between risk factors and disease development, evaluating treatment responses under various timeframes, and identifying patterns of comorbidities. Additionally, data are captured prospectively in real-time or near real-time, thereby limiting the possibility of recall bias. For endometriosis, with its frequent delayed diagnosis and unknown etiology, longitudinal data provide a cache of clues—signs, symptoms, or risk factors—that can be mined to inform guidelines for early diagnosis and to evaluate the long-term outcomes of the disease.

Data patterns indicative of endometriosis have been identified in patients' EHRs several years prior to diagnosis (59). One of these patterns is a temporal link between lower gastrointestinal symptoms with gynecologic pain, with both types of symptoms occurring within 90 days of each other in patients subsequently diagnosed with endometriosis. This type of pattern has important implications for early diagnosis, and as more studies leverage longitudinal EHR data, more combinations of related asynchronous symptoms will likely be identified to provide missing context from what is otherwise considered independent nonspecific symptoms that confound diagnoses.

Beyond symptoms, longitudinal analysis can be extended to evaluate links between endometriosis and modifiable risk factors, such as diet, exercise, or other behavioral or environmental exposures such as smoking and alcohol use, and because longitudinal EHR data can capture changes in behavior, can be used to design studies that resolve reverse causation (60). For example, a study examining the relationship between endometriosis and exercise may show that exercise is associated with less severe symptoms or an absence of disease, implying that exercise acts as an effective treatment or prevention intervention. However, a patient experiencing debilitating pelvic pain is less likely to exercise, so the more probable explanation is the reverse: that endometriosis limits exercise (61). Longitudinal data provide the context required to distinguish between cause and effect. The importance of capturing lifestyle and social determinants of health (SDoH) data in a healthcare setting is well recognized, but the quality of the data is highly inconsistent. Therefore, caution should be used in utilizing this data. Extraction of SDoH variables requires sophisticated algorithms that include natural language processing (NLP). A recently published systematic review demonstrated that among SDoH, smoking, substance, and alcohol use were most commonly extracted features of EHR (62). NLP tools such as cTAKES, CRIS-IE.and Moonstone NLP are among the most popular tools for extracting lifestyle data such as substance use, diet, exercise, and smoking, among others (63–65).

Similarly, ample evidence suggests that as a chronic systemic disease, endometriosis shares both genetic and nongenetic risk factors with associated comorbidities and may influence the incidence of these diseases (6, 22). Endometriosis has a clear genetic basis as several studies have found a higher risk of developing endometriosis among sisters and daughters of women with the disease, indicating a familial clustering of the disease. Twin studies have also provided evidence of a strong genetic component, with concordance rates for endometriosis being higher in monozygotic twins compared to dizygotic twins (66, 67). In addition, biomarkers such as CA125 have been investigated as potential tools for diagnosing endometriosis. However, hey lack sensitivity and specificity for the disease. However, ongoing research in genomics and proteomics may uncover additional biomarkers that could improve the accuracy of endometriosis diagnosis (68, 69).

Women with endometriosis have a significantly increased risk of developing malignancies, such as ovarian, breast, or endometrial cancer, although endometriosis lesions are benign. Women with endometriosis are also at higher risk for hypertension, ischemic heart disease, and myocardial infarction (70, 71). There are gaps in the literature regarding the relationships between endometriosis and comorbidities; many studies are affected by selection bias, confounded by correlated risk factors, or have insufficient follow-up or missing temporal data (7, 22). Longitudinal EHR data can encapsulate complete health histories and thus provide the type of data required to design studies that account for the timing of endometriosis diagnosis relative to other diseases and to identify shared risk factors under the same temporal models. In this way, life-long chronology maps of endometriosis can be generated to identify individuals with endometriosis who are at risk of developing associated comorbidities. Thus, the direct and indirect effects of endometriosis on concomitant and long-term health outcomes can be disaggregated.

## Limitations of electronic health record data

As the reuse of EHR data for research has grown, much progress has been made in understanding the opportunities and limitations accompanying this type of real-world data (72, 73). Some limitations are universal, whereas others will be specific to the research question. As a common and heterogeneous, complex disease that is frequently underdiagnosed and misdiagnosed, endometriosis adds its own nuances to the common challenges of EHR-based research. All of these limitations are further elaborated upon in the subsections below.

### Selection bias and misclassification

Because data collection in EHRs is observational and requires patients to seek care and providers to recognize, diagnose and code disease conditions accurately, one of the biggest challenges of research design with EHR data is selection bias (74). Specifically, for endometriosis, we are concerned with the tendency for diagnoses to be delayed and unevenly distributed among racial or ancestral groups. Delayed diagnosis across the patient pool can mean the patients with a diagnosis have more severe disease, which can lead phenotyping algorithms to select only the most severe cases for inclusion in studies which precludes research on early detection and the stages or subtypes of disease. Underdiagnosis of select groups can lead to a mismatch between the patients being studied and the underlying patient population from which they are drawn perpetuating exclusionary research practices (47). In general, when treatment is delayed in undiagnosed patients, the signs, symptoms, and severity of disease are distorted that can complicate health outcomes research.

Selection bias also plays a role in misclassification, which has implications for study validity. Identification of appropriate controls is imperative in any case-control study design. The likelihood of case contamination in the control population is increased for diseases like endometriosis that have documented patterns of both delayed diagnosis and misdiagnosis. Case contamination can bias estimates for disease-associated risk factors.

Manual chart review remains the gold standard for confirming case-control status and verifying phenotyping algorithms (75). Machine learning tools that assess selection bias and misclassification in EHR-based studies are helpful for bias mitigation (76, 77). Both are prerequisites for using EHRs to study endometriosis.

### Confounder bias

Confounders are the variables that are directly related to both predictor and outcome of interest (78). These variables are used in statistical analyses to estimate the direct effect of predictor while controlling for confounders. EHR data inherently measures confounders that can be used in analyses. For example, in a study to understand the risk of cardiovascular diseases in patients with endometriosis, adjusting for the number of encounters for patients seeking care along with age, BMI/other, and other comorbidities that are potential confounders can be used. However, it is essential to consider that the type of encounter for patients may bias because patients with cardiovascular conditions might be older and have more encounters than patients with endometriosis and no cardiovascular condition. Using variables such as outpatient encounters which refers to patients seeking general care, might resolve the issue of confounding bias (79).

There can also be other confounders, such as smoking, diet, and exercise, that can be inconsistently measured as risk factors in the EHR. Evaluation of methods that control unmeasured confounders such as multiple imputation and propensity score matching or sensitivity analyses that account for unmeasured confounding could help reduce the unmeasured confounding biases (80–82). For endometriosis, multiple imputation and propensity score matching methods would require using a subset of the patient population that has complete data for all measured confounders, whereas sensitivity analyses would help in evaluating the impact of unmeasured confounders such as smoking in the entire patient population without actually controlling for the confounder but instead assessing the change in the conclusions for a study due to unmeasured confounders.

### Collider bias

While accounting for confounders such as the number of visits is informative in a study. It must be noted that a confounder variable might also act as a collider (83). Collider variables are the ones that are related to more than one outcome. In the example of endometriosis and cardiovascular risk, the number of visits could be related to patients who need more care leading to an overrepresentation of endometriosis- cardiovascular relationship. Therefore, stratifying analyses on those in the relationship and those not in the relationship is also essential (83).

### Information bias

EHRs may add noise regarding the time of diagnosis (i.e., the time required to establish the diagnosis). When a patient is entered into an EHR system, clinicians record all new and pre-existing conditions in the form of problem lists (84). Lack of standardization and incompleteness of problem lists could lead to errors in determining the actual time of diagnosis (85). Specifically for endometriosis, a historical diagnosis of endometriosis could be perceived as a new diagnosis at the time it is first entered into the EHR system. This problem is more likely to occur with patients who infrequently access health care (in contrast to those who are seen more frequently). Due to the lack of standardization in diagnostic surgery and deep phenotyping of the disease, it is also challenging to get a more nuanced characterization of patients from structured EHR data alone. More progress can be made by utilizing clinical notes. However, one of the limitations is the quantification of treatment outcomes in the EHR context. 10-point Likert scores or the visual analog scale (VAS) scores for dysmenorrhea, non-menstrual pain, or dyspareunia are not commonly used in routine patient care and would be missed by using EHR data alone.

Complimenting EHR data collection with surveys and patient-reported outcomes using tools such as EPHect surveys for endometriosis could help mitigate information bias and improve study design (86). Thorough phenotyping algorithm,s including natural language processing of unstructured data such as patient notees, ar crucial in designing studies of endometriosis using EHR data (87).

## Challenges and considerations on the use of EHR data for endometriosis research

The use of EHR data for research is a promising approach that can yield valuable insights into the causes and treatments of various health conditions. However, to realize its full potential, addressing certain challenges related to data quality and ethical considerations is crucial.

### Quality of the data

In terms of data quality, EHRs are typically created by healthcare providers and may contain incomplete or inaccurate information, posing a potential threat to the validity and reliability of research findings. As such, it is vital to ensure that data quality checks, such as accuracy testing, are in place before extracting big data for research purposes. Furthermore, it is worth noting that EHRs may not always contain all the necessary data elements needed for specific research, such as detailed family history or information about environmental exposures, which could limit the scope and usefulness of the data. From an ethical standpoint, it is also important to respect patients' privacy rights and ensure that data is collected and handled in a manner that is compliant with applicable laws and regulations.

### Ethical considerations and issues

One potential issue with using EHR data for research is that it may result in the potential violation of patients' privacy. EHR data often contain sensitive personal information, such as a patient's medical history, diagnostic test results, and treatment details. If this information is not handled carefully, it could be accessed by unauthorized individuals or used for purposes other than research, which could potentially violate patients' privacy and lead to legal and ethical issues. Another issue is the question of what to do with incidental findings, which are unexpected or unanticipated results that are discovered during genomic research. These findings can have significant implications for the health and well-being of research participants, but there is no consensus on how to handle them. Some researchers argue that incidental findings should be disclosed to participants, while others argue that this could cause unnecessary anxiety and should only be disclosed if there is a clear medical benefit.

### Local and global challenges

Endometriosis researchers who use electronic health records (EHR) and their patients may face several local and global challenges in terms of personal integrity and data commodification. One local challenge that endometriosis researchers using EHR may face is obtaining informed consent from patients to use their medical information in research. To use EHR in research, individuals must give explicit permission for their medical information to be used in this way. This can be a challenging process, as it requires researchers to explain the purpose and potential benefits of the research clearly, as well as the risks and limitations, to obtain valid and informed consent from patients. Another local challenge that endometriosis researchers using EHR may face is protecting the privacy of patients and their medical information. The use of EHR in research involves the collection and use of sensitive personal medical information, which must be protected from unauthorized access or disclosure. Researchers must implement appropriate measures, such as the deidentification of data, to safeguard the privacy of patients and ensure that their personal information is used only for the purposes for which it was collected.

On a global level, endometriosis researchers using EHR and their patients may face challenges related to the commodification of data. This refers to the use of personal data for commercial gains, such as by selling it to third parties or using it to develop and sell products or services. In recent years, there has been a growing trend toward the commercialization of personal data, with companies collecting and selling individuals' personal information for a variety of purposes. This raises concerns about the potential for individuals' personal information to be used for purposes that they did not consent to or to be exploited for financial gain without their knowledge or consent.

Another major challenge in conducting electronic health record (EHR)-based research on a global scale is the lack of standardization in the terminology and data elements used in EHR systems. In many cases, different hospitals, clinics, and countries use different terms and codes to describe the same medical conditions, procedures, and measurements. This can make it difficult to compare and combine EHR data from different sources, leading to inconsistencies and biases in research findings. To address this challenge, some researchers have proposed the use of generalizable predictive models, which are algorithms that can be trained on data from one population and applied to data from another population (88). While these models have the potential to improve the generalizability of EHR-based research, they should be used with caution so they do not exacerbate the health disparities. If the data used to train the model does not accurately represent the population of interest, the predictions made by the model may be inaccurate or biased. In conclusion, the lack of standardization in terminology and data elements in EHRs is a global challenge in EHR-based research. Researchers should be aware of this issue and use generalizable predictive models with caution to avoid introducing biases and inconsistencies into their findings.

To address these issues and challenges, endometriosis researchers using EHR can take a number of steps, including obtaining explicit and informed consent from patients to use their medical information in research, implementing appropriate safeguards to protect the privacy of patients and their medical information, being transparent about how the data collected will be used and shared and carefully considering the representativeness and biases in the data used to train models to ensure that their predictions are accurate and fair. Additionally, researchers can advocate for strong data protection laws and regulations to prevent the unauthorized use or exploitation of personal data. Overall, using EHR data for research is complex and presents a number of challenges, including issues around data quality, the availability of relevant data elements, and the ethical considerations around incidental findings. It is important for researchers to carefully consider these issues when designing and conducting research using EHR data. Additionally, addressing the challenges associated with personal data commodification in endometriosis research using EHR requires a collaborative effort between researchers, patients, policymakers, and the broader community. By working together and advocating for strong privacy protections, we can help to ensure that personal data is used ethically and responsibly and that individuals retain control over their own data.

## Future outlook

EHRs are continuously growing, and researchers are continuously improving the algorithms that make sense of this data. Electronic phenotyping will become better and deeper as algorithms learn to incorporate the dynamic relationships among clinical variables in their predictions (58). Furthermore, as clinical notes become easier to parse, automated extraction of useful information from clinical notes, without loss of context or relevant detail, is an increasingly active area of natural language processing research (89, 90). Concepts extracted from the notes provide an invaluable layer of information for studies of heterogeneous diseases, especially studies incorporating social and behavioral determinants of health; these additional data can be the defining line between speculation and regulatory-grade real-world evidence (91). As EHRs continue to expand, the integration of genetics and diversity will be crucial for improving the accuracy and usefulness of data analysis. With advancements in algorithms and electronic phenotyping, it will be possible to incorporate more genetic data into EHRs, enabling researchers to better understand the genetic determinants of complex diseases like endometriosis. Genetic data can provide insights into the underlying causes of endometriosis, such as heritable genetic mutations or epigenetic changes. In addition, by including data from diverse populations, researchers can identify potential disparities in disease risk and outcomes across different groups. This can help to develop more tailored treatment plans and interventions that account for differences in genetics, culture, and lifestyle. Furthermore, EHR-based research can provide a unique opportunity to investigate how genetic and environmental factors interact to contribute to disease risk, which can inform targeted prevention strategies. Incorporating genetics and diversity into EHR-based research has the potential to revolutionize our understanding of complex diseases like endometriosis and improve health outcomes for patients (29, 36).

Patient-generated data will also become a standard part of EHRs. Self-tracking of signs and symptoms of endometriosis through mobile devices and digital technologies has already proven useful for collecting more complete data and characterizing subtypes of the disease (8, 40). The greatest strides will be made when data and algorithmic advances converge in studies across widely distributed networks of healthcare providers. Longitudinal, multicenter studies are the key to building portable pipelines that can stratify patients with heterogeneous diseases to identify risk factors and predict long-term trajectories (92). Additionally, as EHRs begin to incorporate more patient-generated data, it will be important to ensure that the data collected is representative of diverse populations. The inclusion of data on social and behavioral determinants of health can help to identify disparities in disease risk and outcomes across different populations. Longitudinal, multicenter studies that include diverse patient populations will be essential for building predictive models that can accurately identify risk factors and forecast long-term outcomes for patients with endometriosis and other complex diseases (93).

## Conclusions

In conclusion, the use of EHRs for endometriosis research is a valuable tool that has the potential to benefit patients of all racial and ethnic groups. Currently, there are significant racial disparities in endometriosis research, with a limited representation of minority groups in clinical trials and research studies. By expanding the use of EHRs for endometriosis research, we can overcome some of these barriers and ensure that all patients have access to the latest advancements in diagnosis and treatment.

Moreover, EHR-based research can help identify potential risk factors and preventative measures for endometriosis. By analyzing large-scale, retrospective data from diverse populations, we can identify trends and risk factors that may not have been apparent in smaller, more homogenous studies. This can help improve the accuracy of early diagnosis and enable clinicians to provide more personalized care to patients based on their unique risk factors.

Finally, EHR-based research can also help us understand the potential long-term health consequences of endometriosis in patients of all racial and ethnic groups. By collecting and analyzing data from a wide range of patients, researchers can identify potential comorbidities, complications, and long-term effects of endometriosis. This information can be used to develop targeted interventions that address the specific needs of patients based on their individual risk factors and health history.

Overall, the expansion of EHR-based research has the potential to transform our understanding of endometriosis and improve outcomes for patients of all racial and ethnic groups. By working to ensure that EHR data is high-quality and collected, and handled in an ethical and responsible manner, we can unlock the full potential of this powerful tool and improve health outcomes for all patients.
